# The impact of vitamin D on the etiopathogenesis and the progression of type 1 and type 2 diabetes in children and adults

**DOI:** 10.3389/fendo.2024.1360525

**Published:** 2024-04-08

**Authors:** Candong Li, Jiaowen Fu, Yipeng Ye, Junsen Li, Yangli He, Tuanyu Fang

**Affiliations:** ^1^ Department of Endocrine, Hainan General Hospital, Hainan Affiliated Hospital of Hainan Medical University, Haikou, Hainan, China; ^2^ Department of Health Care Centre, Hainan General Hospital, Hainan Affiliated Hospital of Hainan Medical University, Haikou, China

**Keywords:** vitamin D, diabetes mellitus, diabetic complications, insulin resistance, pathogenesis

## Abstract

Diabetes is a common chronic metabolic disease with complex causes and pathogenesis. As an immunomodulator, vitamin D has recently become a research hotspot in the occurrence and development of diabetes and its complications. Many studies have shown that vitamin D can reduce the occurrence of diabetes and delay the progression of diabetes complications, and vitamin D can reduce oxidative stress, inhibit iron apoptosis, promote Ca^2+^ influx, promote insulin secretion, and reduce insulin resistance. Therefore, the prevention and correction of vitamin D deficiency is very necessary for diabetic patients, but further research is needed to confirm what serum levels of vitamin D_3_ are maintained in the body. This article provides a brief review of the relationship between vitamin D and diabetes, including its acute and chronic complications.

## 1 Introduction

Diabetes mellitus (DM) is a group of metabolic diseases characterized by chronic hyperglycemia, caused by defects in insulin secretion and/or utilization. Over the past 30 years, the prevalence of diabetes in China has significantly increased. A thyroid iodine nutrition status and diabetes epidemiological survey conducted by the Endocrinology Branch of the Chinese Medical Association from 2015 to 2017 showed that the prevalence of diabetes among the Chinese population aged 18 and above was 11.2% (1). Vitamin D, a fat-soluble vitamin, not only plays a role in calcium and phosphorus regulation but is also closely related to diabetes, cardiovascular diseases, immune regulation, tumors, muscle strength enhancement, and fall prevention. Studies have shown that vitamin D is closely related to pancreatic function, immunity, genetic polymorphism, and the occurrence of diabetic complications. In recent years, the relationship between vitamin D and the development of diabetes has become a research focus in the field of diabetes. This article provides a brief overview of the effects of vitamin D on the pathogenesis and progression of complications in different types of diabetes.

## 2 Overview of vitamin D

**Figure 1:**
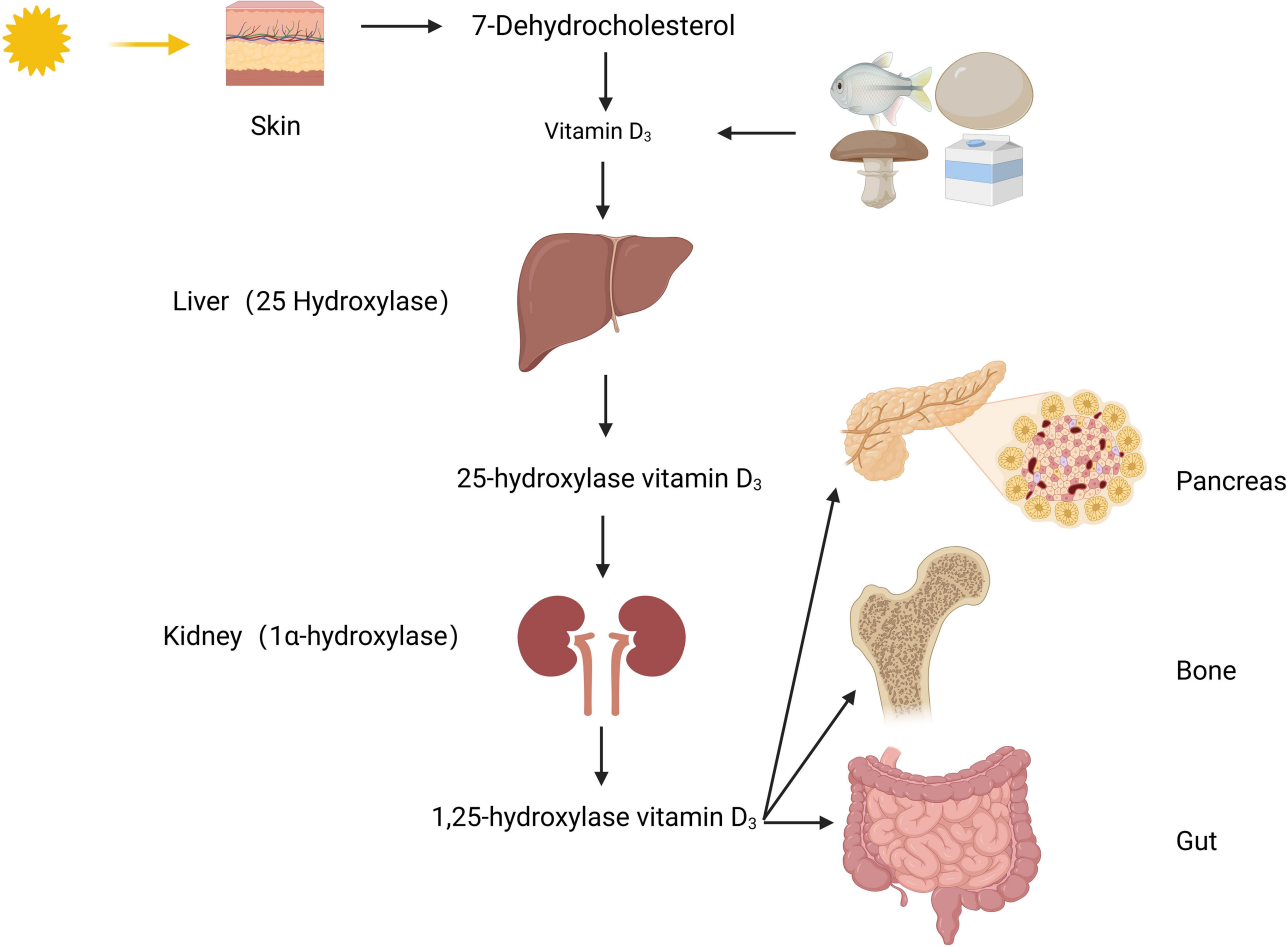
Metabolic pathways of vitamin D.

Vitamin D, a derivative of steroids, belongs to the cyclopentane polyhydrophenyl compound class. It is chemically stable, except for being light-sensitive. There are two main sources of vitamin D: one is converted from 7-dehydrocholesterol in the skin under the influence of ultraviolet light; the other is from vitamin D_2_ in mushrooms exposed to sunlight and vitamin D3 in foods such as liver, milk, and cod liver oil. The vitamin D_2_ and D_3_ obtained from these sources are inactive forms, and they cannot be converted into each other, collectively referred to as vitamin D. To obtain biologically active 1,25(OH)_2_D_3_, it needs to undergo two hydroxylations in the body (**Figure 1**). Firstly, the inactive vitamin D is converted to 25(OH)D_3_ in the liver under the catalysis of the 25-hydroxylase enzyme. 25(OH)D_3_ is the main storage form in the body, and its level reflects the nutritional status of vitamin D. Then, 25(OH)D_3_ is further converted to 1,25(OH)_2_D_3_ in the kidneys under the action of 1α-hydroxylase. 1,25(OH)_2_D_3_ binds to vitamin D receptors(VDR) widely present in tissues and exerts its effects in the body (2).

## 3 Relationship between vitamin D levels and diabetes

### 3.1. Vitamin D and diabetes

A number of studies have been conducted in the population to explore the relationship between vitamin D_3_ and glycemic control (**Table 1**). Serum 25(OH)D_3_ levels have been shown to have a negative dose-response correlation with the risk of type 2 diabetes mellitus (T2DM) (3), and vitamin D supplementation reduces the risk of T2DM (4), decreases the risk of T2DM in pre-diabetic patients, and increases the chances of restoring normal glucose tolerance (5). However, the benefits of vitamin D_3_ for the prevention of T2DM may be limited to non-obese subjects (5) or patients with vitamin D deficiency (6).

**Table 1:** The relationship between vitamin D and diabetes.

Reference	Year	Sample size	Research Design	Conclusion of the study
(3)	2020	6940	Cohort studies	In subjects with healthy sleep patterns, the higher the serum 25(OH)D_3_ concentration, the lower the risk of developing T2DM.
(4)	2022	2423	Randomized clinical trials (RCT)	Supplementing with 4,000 IU of vitamin D per day can reduce the risk of diabetes. Vitamin D had a small beneficial effect on change in fasting plasma glucose.
(5)	2020	4896	Meta-analysis	Vitamin D supplementation reduces the risk of T2DM in participants with prediabetes. Reversion of prediabetes to normoglycemia was significantly increased by vitamin D supplementation. The benefit of the prevention of T2DM appears to be confined to nonobese subjects.
(6)	2019	2423	Clinical trial (CT)	Among persons at high risk for T2DM not selected for vitamin D insufficiency, vitamin D supplementation at a dose of 4000 IU per day did not result in a significantly lower risk of diabetes than placebo.
(7)	2023	1074	A cross-sectional study	In the T2DM patient cohort, the mean blood 25(OH)D_3_ levels were 17.05 ng/ml. In comparison to the winter and spring, both males and females showed higher 25(OH)D_3_ levels in the summer. HbA1c and vitamin D levels were negatively correlated.
(8)	2021	130	RCT	Oral daily doses of vitamin D improve HbA1c levels over the 3-month and 6-month period, followed by a significant decrease in advanced oxidation protein products levels over the 3-month period when higher vitamin D doses are given.
(9)	2021	1932	Meta-analysis	Vitamin D supplementation significantly improved fasting blood glucose, postprandial blood glucose, and quantitative insulin sensitivity check index in diabetes and prediabetes with baseline 25(OH)D_3_<30 ng/ml. Higher percentages regressing from prediabetes to normal glucose status and lower percentage progressing from prediabetes to diabetes were found in the supplementation group. The positive effects of vitamin D supplementation on body mass index, waist, HDL-C, LDL-C, and CRP were also demonstrated.

T2DM, type 2 diabetes mellitus.

Achieving blood glucose control is one of the goals of diabetes treatment. Serum 25(OH)D _3_ levels can be an independent risk factor for increased levels of glycated hemoglobin in T2DM, with women being at a higher risk of vitamin D deficiency (7). In addition to finding that vitamin D can be a risk factor for glycemic control, it is interesting to note that in patients with T2DM, the combined administration of metformin and vitamin D resulted in better glycemic and glycosylated hemoglobin control compared to metformin alone (8). The efficacy of vitamin D on glycemic stability and insulin function was also found in patients with prediabetes (9). Similar results were obtained in animal experiments. Vitamin D supplementation reduced blood glucose, insulin levels, and improved insulin resistance (IR) in rats in a prediabetic model, and this efficacy was proportional to the dose of vitamin D supplementation (10). The above findings suggest that vitamin D helps in blood sugar control. However, there is still a need for larger studies in the future to examine the dose, duration, and most appropriate population for vitamin D supplementation to determine the relationship between vitamin D and glycemic control in diabetes. Serum 25(OH)D_3_ insufficiency is closely related to the development of type 1 diabetes mellitus (T1DM) in children (11).

### 3.2 Vitamin D and diabetic complications

**Figure 2:**
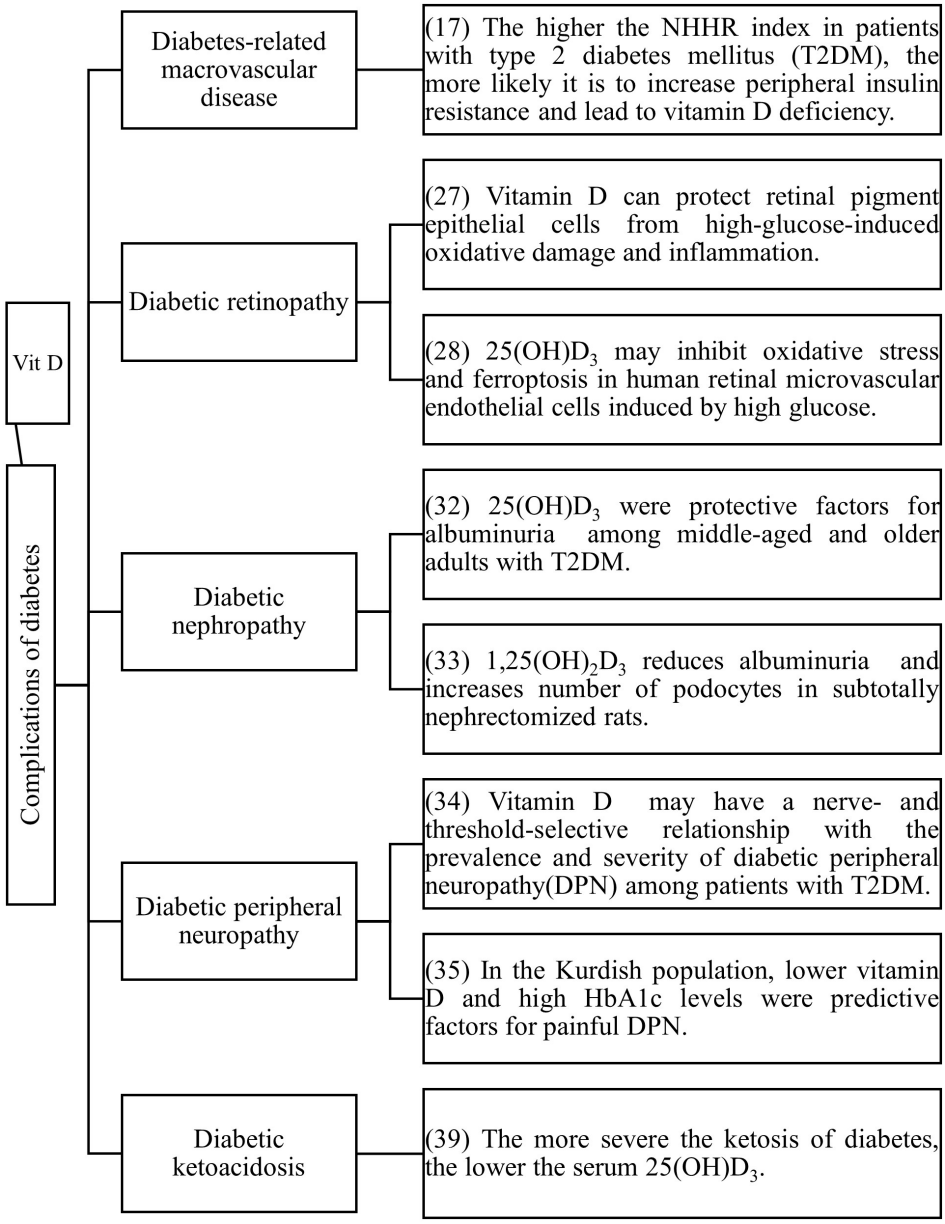
The relationship between vitamin D and diabetic complications.

Vitamin D is not only related to the occurrence and development of diabetes, but numerous studies have also confirmed that vitamin D deficiency is closely associated with diabetic complications (**Figure 2**).

Although vitamin D does not reduce all-cause mortality in older adults (12). However, there are still a series of studies proving that vitamin D can prevent cardiovascular risk in people with diabetes. The incidence of cardiovascular diseases in T2DM patients increases by 2 to 3 times (13), and a follow-up study of T2DM patients confirmed that vitamin D deficiency is the strongest correlating factor for the occurrence of cardiovascular diseases in these patients (14). Vitamin D deficiency is also related to endothelial dysfunction and atherosclerosis (15, 16). The non-high-density lipoprotein cholesterol/high-density lipoprotein cholesterol ratio (NHHR) is a new comprehensive index of atherosclerotic lipids (17). NHHR is negatively correlated with vitamin D in T2DM patients (18). A higher NHHR index indicates a greater tendency for peripheral cholesterol deposition, and an increased distribution of cholesterol to the periphery may lead to IR or pancreatic β-cell dysfunction (19), resulting in vitamin D deficiency. Supplementing vitamin D can slow down the progression of myocardial dysfunction in T2DM patients without complications (20).

A prospective study on T2DM patients found that higher serum 25(OH)D_3_ levels can reduce the risk of diabetic retinopathy, diabetic nephropathy, and diabetic neuropathy, and serum 25(OH)D_3_ levels within a certain range (10-106 nmol/L) have a linear dose-response relationship with diabetic retinopathy and diabetic nephropathy (21). Inflammation has been shown to contribute to the occurrence and development of diabetic retinopathy (22–27). The pathogenesis of diabetic retinopathy is related to inflammation and fibrosis (23), and it has been reported that the production of pro-inflammatory cytokines such as IL-1, TNF-α, and VEGF increases in the vitreous body of patients with diabetic retinopathy and in animal models of the retina (22, 24). High glucose-induced upregulation of pro-inflammatory cytokines can lead to the destruction of the blood-retinal barrier (BRB), cell death, and angiogenesis (22, 25). Supplementing vitamin D can protect retinal epithelial cells from high glucose-induced oxidative stress, inflammation and ferroptosis which may be one of the mechanisms by which vitamin D prevents the progression of diabetic retinopathy (28, 29).

Diabetic nephropathy is a major cause of disability and death in middle-aged and elderly patients with T2DM, significantly affecting their quality of life and safety. Progressive proteinuria and renal function deterioration are the main clinical symptoms of diabetic nephropathy (30–32). A study on elderly T2DM patients in China found that the incidence of vitamin D deficiency was significantly higher in patients with proteinuria than in those without (33). In animal experiments, vitamin D can prevent podocyte damage, thereby reducing proteinuria and glomerulosclerosis (34).

Peripheral nerve damage is also a common complication of diabetes mellitus. Patients lacking vitamin D are more likely to experience nerve function deficits associated with diabetic peripheral neuropathy than those with sufficient vitamin D (35). Among the Kurdish population, lower levels of vitamin D and higher levels of HbA1c are predictive risk factors for painful diabetic peripheral neuropathy (36). Controlling blood glucose alone cannot prevent the progression of peripheral neuropathy in T2DM. Chen T (37) found that monthly intramuscular injections of high-dose vitamin D improved peripheral neuropathy. This may provide new ideas for treating diabetic peripheral nerves. However, more research is needed to determine the exact course of treatment and dosage. In a study of diabetes mellitus in children (38), children with vitamin D deficiency did not complain of peripheral neuropathy, but sensory nerve action potential of sural nerve and motor peroneal nerve velocity were statistically significantly lower in diabetic patients with vitamin D deficiency compared to diabetic patients with normal vitamin D levels.

Diabetic ketosis is one of the acute complications of diabetes mellitus. Serum 25(OH)D_3_ levels are lower in ketosis-prone T2DM compared to non-ketosis-prone T2DM (39, 40), and serum 25(OH)D_3_ levels are related to the severity of pancreatitis concurrent with diabetic ketoacidosis (41).

## 4 Vitamin D and the pathogenesis of diabetes

### 4.1 Vitamin D and pancreatic function

**Figure 3:**
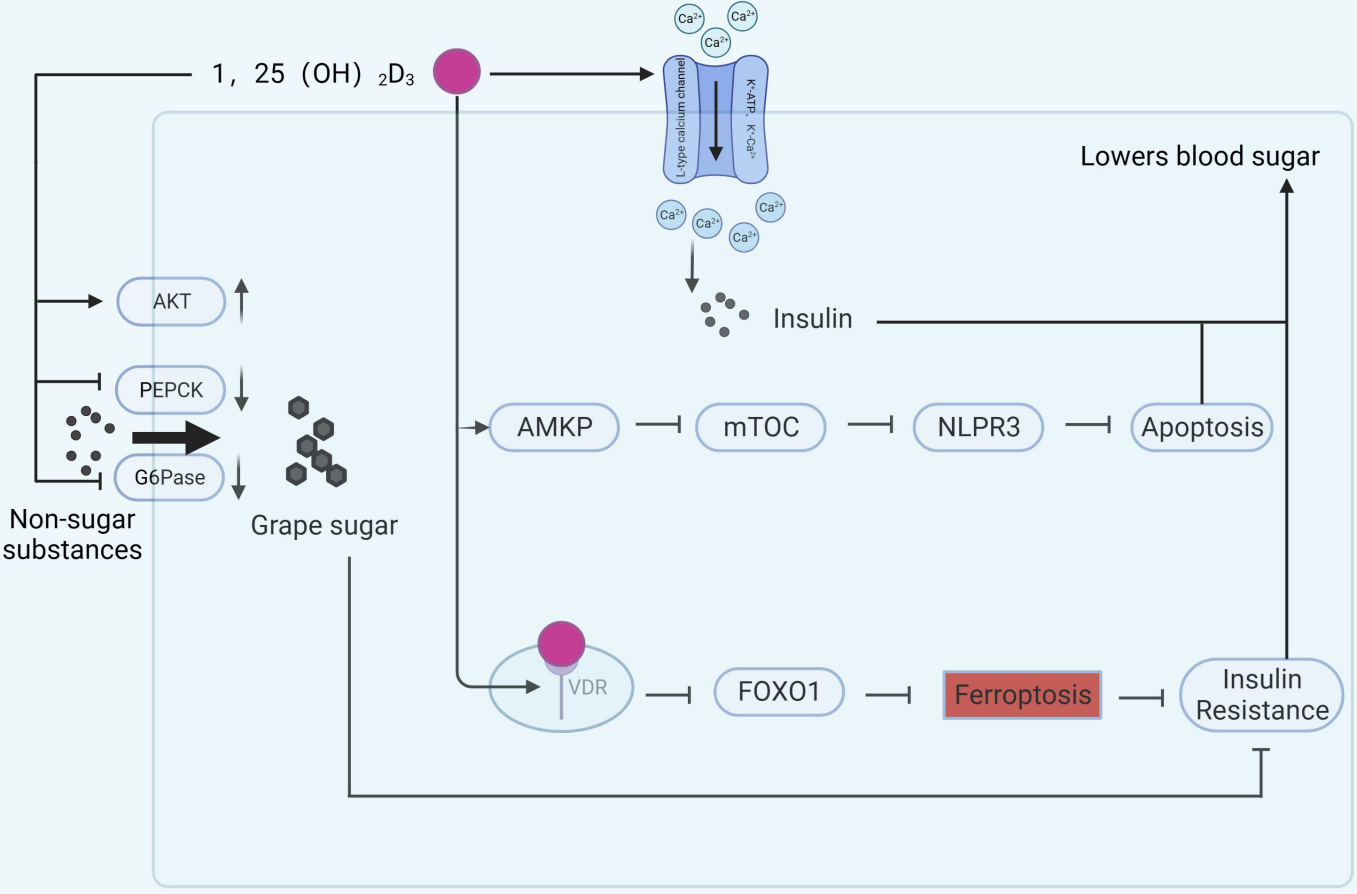
Mechanisms of vitamin D involvement in pancreatic function. (AKT, protein kinase B; PEACK, phosphoenolpyruvate carboxykinase; G6Pase, Glucose-6-phosphatase; AMPK, Adenosine 5’-monophosphate (AMP)-activated protein kinase; mTOR, mammalian target of rapamycin).

Vitamin D may affect pancreatic function through several signaling pathways (**Figure 3**). Glucose-6-phosphatase (G6Pase) and phosphoenolpyruvate carboxykinase (PEPCK) are two key enzymes that convert non-carbohydrate substances into glucose (42, 43). Their abnormal expression is closely associated with enhanced gluconeogenesis and considered a marker for T2DM (44). Combined treatment with vitamin D and aerobic exercise can upregulate protein kinase B (AKT) in liver cells of T2DM rats, downregulate PEPCK and G6Pase expression, improve liver function, and alleviate IR (45). Vitamin D-binding protein (VDBP) is the primary plasma carrier maintaining vitamin D and its metabolites. Deficiency of VDBP may lead to pancreatic α-cell atrophy and proliferation, alters Na^+^ channel conductance, reduces cellular activation by glucose, and decreases the rate of glucagon secretion *in vivo*, potentially increasing the incidence of late-onset T1DM (46). A double-blind, randomized, controlled clinical trial (47) showed that vitamin D supplementation improved β-cell function in patients with serum 25(OH)D_3_ levels below 12 ng/mL compared to placebo.

Intraperitoneal injection of 1,25(OH)_2_D_3_ treats dexamethasone-induced IR in rats and improves islet function. The reason is related to the alteration of calcium ions after activation of L-type voltage-dependent calcium channel (VDCC), K^+^-ATP, K^+^-Ca^2+^, and Kv channels by 1,25(OH)_2_D_3_, followed by activation of downstream PKC, PKA, and so on, which promotes insulin secretion (48). *In vitro* studies indicate that vitamin D can activate AMP-dependent protein kinase, inhibit the mammalian target of rapamycin (mTOR) pathway, thereby inhibiting the activation of the NLRP3 inflammasome and reducing β-cell apoptosis, promoting insulin release (49). Vitamin D may also reduce β-cell apoptosis in T2DM by inhibiting nuclear factor κB and downregulating the expression of divalent metal transporter 1 (DMT1) , alleviating pancreatic iron overload (50).

Ferroptosis, a newly discovered form of cell death, is considered a crucial factor in the pathogenesis of many inflammatory diseases (51). Ferroptosis is also considered a new target for diabetes (52). In the rat diabetes model, ferroptosis-related indicators such as GPX4 and SLC7A11 were downregulated (53) and ACSL4 was upregulated (54). 1,25(OH)_2_D_3_ treatment not only lowered blood glucose, but also reversed the changes in the above metrics. The mechanism is related to 1,25(OH)_2_D_3_/VDR/FOXO1. Binding of 1,25(OH)_2_D_3_ to the VDR inhibits ferroptosis in pancreatic β-cells and ameliorates IR by down-regulating FOXO1 expression (54). Vitamin D induces cellular autophagy while inhibiting streptozotocin-induced β-cell apoptosis, increasing insulin secretion and increasing β-cell resistance to cellular stresses encountered in hyperglycemic states (55). Excessive or prolonged exposure to nitric oxide leads to β-cell dysfunction, whereas vitamin D induces and maintains high levels of the A20 gene protein and reduces nitric oxide levels, thus serving to protect β-cell function (56).

### 4.2 Vitamin D and immune function

**Figure 4:**
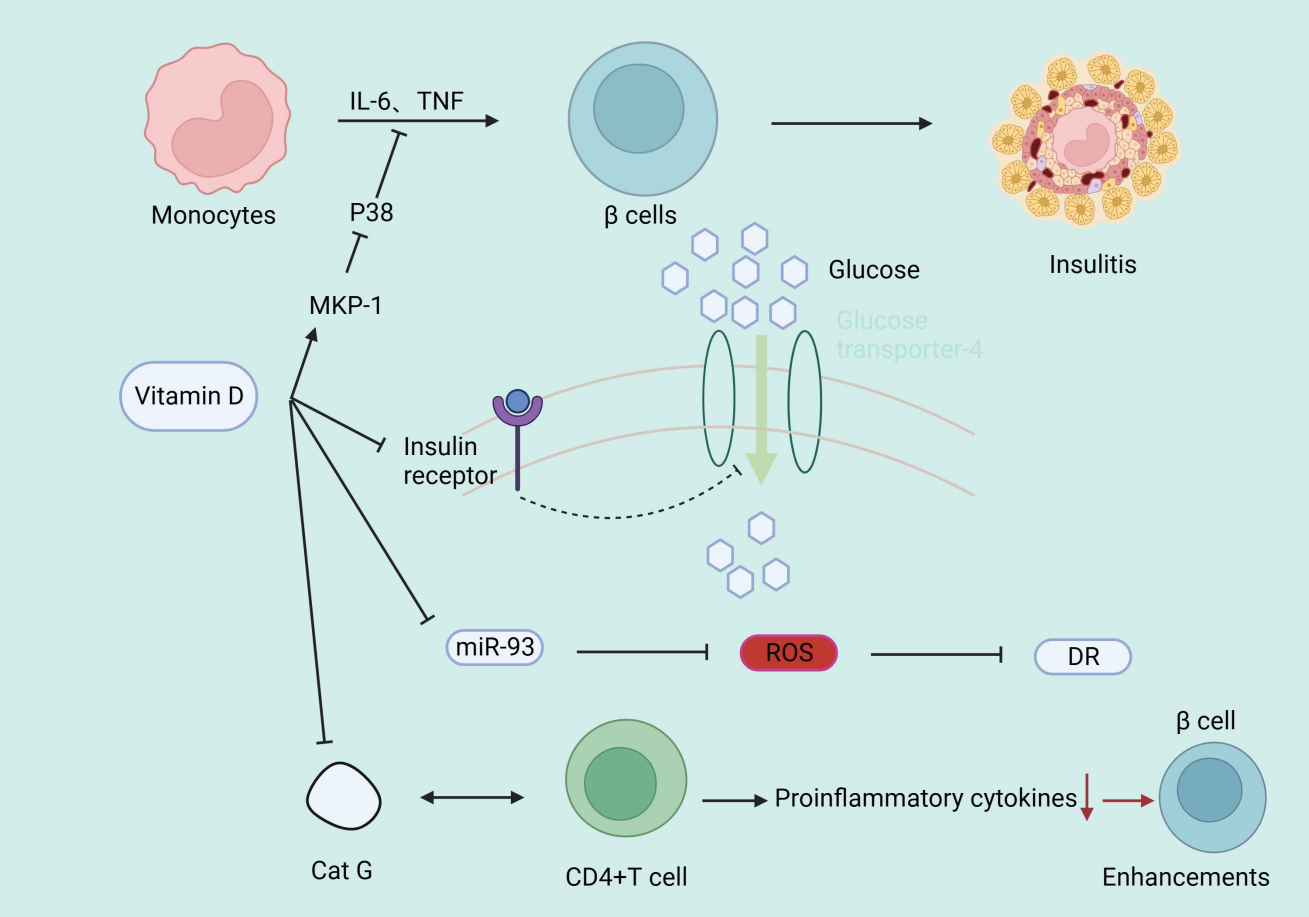
Vitamin D is involved in diabetes through immunization.

Type 1 diabetes is an autoimmune disease. Vitamin D, by binding with its receptor, reduces pro-inflammatory cytokines in immune cells and has an immunomodulatory effect (57, 58). CD4+ T lymphocytes are the primary immune-mediated cells in the development of T1DM (59). Vitamin D supplementation can downregulate cathepsin G (Cat G) expression, hindering CD4+ T lymphocyte activation, thus enhancing pancreatic β-cell function (60). Systemic lupus erythematosus (SLE) is an immune disease. It has been found that IR is more prevalent in SLE patients than in controls (61), while serum vitamin D is negatively correlated with CD4+/CD8+ T cells (62), IFN-α levels (63), IL-17, IL-23 (64) in SLE patients. 25(OH)D_3_ is further converted into 1,25(OH)_2_D_3_ in the kidneys by 1α-hydroxylase, which is expressed by antigen-presenting cells (65), indicating an association of the immune microenvironment with active vitamin D production, suggesting that vitamin D may mediate the disease process in T1DM. Complement can trigger the contraction of CD4+ type 1 helper T cell responses by inducing the intrinsic expression of VDR and vitamin D-activating enzyme CYP27B1, enabling T cells to both activate and respond to vitamin D (66). Vitamin D deficiency can epigenetically suppress Jarid2 expression in hepatic stellate cells (HSCs) and activate the Mef2/PGC1a pathway, leading to fat macrophage infiltration. Macrophages secrete MiR-106b-5p, inducing downregulation of the PIK3CA/PIK3R1/PDPK1/AKT signaling pathway, promoting fat IR (67). Vitamin D supplementation can reduce IR in diabetic rats by lowering phosphorylation levels of insulin receptor substrate 1 (IRS1), leading to impaired glucose transporter 4 and reduced glucose uptake, as well as increasing peroxisome proliferator-activated receptor γ expression and reducing nuclear factor κB phosphorylation levels (10). In addition, vitamin D increases insulin secretion and sensitivity by up-regulating mitogen-activated protein kinase phosphorylase-1 (MKP-1), inhibiting lipopolysaccharide-induced p38 phosphorylation, suppressing the production of IL-6 and TNF-α in human monocytes (68), and increasing the activity of the antioxidant system (69). Vitamin D has a strong protective effect on chronic kidney diseases (70, 71). The possible mechanism is that 1,25(OH)_2_D_3_ reduces oxidative stress by increasing renal antioxidant capacity, inhibiting hyperglycemia-induced cell apoptosis, preventing podocyte damage, promoting anti-inflammatory action, and improving endothelial function (72–74) (**Figure 4**). In T1DM, some patients may experience a clinical remission period, also known as the “honeymoon phase”, after receiving insulin therapy in the early stage of the disease, in which the islet function of the patient may partially or completely return to normal levels, and vitamin D may reduce the concentration of serum TNF-α through immunomodulatory effects, thereby reducing the inflammatory response during the honeymoon phase and prolonging the duration of the honeymoon phase (75), but the amount of vitamin D supplementation needs to be further studied.

### 4.3 Vitamin D polymorphism and diabetes

Polymorphism in the VDR gene plays a role in the control and progression of T1DM, with higher levels of vitamin D providing protection for pancreatic cells (76). Polymorphisms in the 25-hydroxylase (CYP2R1) gene, rs12794714 and rs10766196, are associated with a higher risk of T1DM (77). It was found that CYP2R1 mRNA expression in the livers of mice fed a high-fat diet was significantly lower than in those fed a low-fat diet (78). As well, an activity analysis of the isolated liver showed that obese mice produced significantly less 25(OH)D_3_ than lean mice, indicating that reduced circulating 25(OH)D_3_ is partly due to the decreased expression of CYP2R1 in obese mice. The onset of T1DM might also be related to polymorphism in the CYP27B1 gene located on chromosome 12, where polymorphisms in the CYP27B1 gene could lead to reduced levels of 1α-hydroxylase, thereby affecting the conversion of vitamin D to 1,25(OH)_2_D_3_ and increasing the susceptibility to T1DM (79). Tangjittipokin (80) found that VDR gene-related variations of ApaI (rs7975232), TaqI (rs731236), and BsmI (rs1544410) were negatively associated with vitamin D and IL-10 levels in children with T1DM. Alleles of DHCR7, GC, CYP2R1, and CYP24A1 play a synergistic role in susceptibility to type 1 diabetes by functioning in the vitamin D pathway and serum vitamin D levels (81). A genome-wide association Meta-analysis study by Jiang X et al. (82) of 79,366 European individuals suggests that CYP24A1 (rs17216707) is negatively correlated with 25(OH)D_3_ levels. The correlation between VDR gene rs739837 polymorphism and susceptibility to T2DM and gestational diabetes mellitus (GDM) (83) pointed out that the VDR gene rs739837 polymorphism is significantly correlated with susceptibility to T2DM. Studies in gestational diabetes mellitus (84, 85) confirmed that single nucleotide polymorphisms (SNPs) mutations at VDR-rs10783219 and MTNR1B-rs10830962 significantly increased the risk of GDM, ApaI-rs79785232, BsmI-rs1544410, FokI-rs2228570 and TaqI-rs731236 are associated with GDM occurrence in the Saudi Arabian region (**Table 2**).

**Table 2:** Effects of vitamin D and genes on diabetes.

Literature	Gene	SNPs
(78)	CYP2R1	rs12794714 rs10766196
(80)	CYP2R1 CYP27B1	- -
(81)	ApaI TaqI BsmI	rs7975232 rs731236 rs1544410
(82)	DHCR7 GC CYP2R1 CYP24A1	rs12785878 rs2282679 rs2060793 rs6013897
(83)	CYP24A1	rs17216707
(84)	VDR	rs739837
(85)	VDR MTNR1B	rs10783219 rs10830962
(79)	ApaI BsmI FokI TaqI	rs79785232 rs1544410 rs2228570 rs731236

## 5 Conclusion and prospects

In recent years, from D to D, the role of vitamin D in the occurrence and development of diabetes has gained attention. The relationship between vitamin D and the onset, progression of diabetes, the ideal daily dosage of vitamin D supplementation, and the optimal serum 25(OH)D_3_ levels for maximum benefits in diabetes risk individuals, early-stage patients, blood sugar control, and diabetes-related complications still require more reliable clinical studies and basic experiments for confirmation. Current research suggests that moderate supplementation of vitamin D can improve the onset and progression of diabetes and its complications, but routine large-dose supplementation is not recommended. It is believed that the relationship between the two will be further verified in the near future.

## Author contributions

CL: Conceptualization, Writing – review & editing. JF: Writing – original draft. YY: Writing – review & editing. JL: Writing – review & editing. YH: Conceptualization, Supervision, Writing – review & editing. TF: Conceptualization, Supervision, Writing – review & editing.
